# Colorectal ESD in day surgery

**DOI:** 10.1007/s00464-016-5407-7

**Published:** 2017-02-08

**Authors:** Tomohiko Ohya, Richard Marsk, Klas Pekkari

**Affiliations:** 1 Department of Endoscopy, The Jikei University School of Medicine, Tokyo, Japan; 2 Department of Clinical Sciences, Danderyd Hospital, Karolinska Institutet, Stockholm, Sweden; 3 Department of Surgery and Urology, Danderyd Hospital, 182 88 Stockholm, Sweden

**Keywords:** ESD, Colorectal, Day surgery, EMR, Endoscopy

## Abstract

**Background:**

Colorectal endoscopic submucosal dissection (ESD) was developed in Japan and is growing in popularity in Europe. Patients undergoing a colorectal ESD procedure in Japan are hospitalized for several days. In this study, we investigated the feasibility of colorectal ESD as an outpatient procedure in a European setting.

**Methods:**

A prospective cohort of all patients undergoing colorectal ESD at Danderyds Hospital, Stockholm, Sweden from April 2014 to December 2015 were studied. Data on patient demographics, procedural outcome and 30-day readmissions were studied. Data are presented as median (range), mean ± SD or true numbers as appropriate.

**Results:**

A total of 182 patients underwent a colorectal ESD during the study period. Of the 182 these, 11 were scheduled for an in-hospital procedure and of 171 patients scheduled for a day-procedure and 15 were admitted for observation. The remaining 156 patients were discharged after 2–4 h of observation and comprise the study cohort. Mean age was 69 years. Median lesion size was 28 (10–120) mm, and median resection time was 65 (10–360) min. Lesions were located as follows: anal canal 1 (0.6%), rectum 52 (33.3%), sigmoid 17 (10.9%), descending 3 (1.9%), transverse 24 (15.4%), ascending 29 (18.6%), and cecum 30 (19.2%). Eight (5.1%) of the 156 day surgery patients returned for medical attention during the postoperative 30-day period. Three of them were admitted for in-hospital observation. None of the day surgery patients required any surgical intervention.

**Conclusion:**

Uncomplicated colorectal ESD can safely be carried out in a day surgery setting.

Colorectal cancer is the third most common cause of cancer-related death in Sweden [1]. The pathogenesis is described by the adenoma–carcinoma sequence where neoplastic polyps are precursors to invasive submucosal cancer in colon and rectum [2]. In many European countries, including Sweden, colorectal cancer screening programs have been implemented. These screening programs lead to increased detection rates of precancerous adenomas as well as superficial carcinomas. Removal of these lesions lowers the incidence of colorectal cancer [3–5].

Endoscopic removal of stalked polyps can be easily managed with snare polypectomy. Smaller sessile and flat adenomas can be removed en bloc with endoscopic mucosal resection (EMR). Larger adenomas (>20 mm in diameter) are difficult to remove en bloc with EMR [6, 7]. The options for removal of these lesions are endoscopic piecemeal mucosal resection (EPMR) or endoscopic submucosal dissection (ESD). EPMR is considered as a time-efficient method with a short learning curve and with low complication rates. The drawbacks of EPMR are that it is difficult for the pathologist to assess the depth of the lesion and to ensure microscopic radical removal. High recurrence rates are seen after EPMR, in some series up to 12–26% [8, 9].

In contrast, ESD enables en bloc resection of lesions regardless of size with low recurrence rates [10]. In addition, the pathologist receives a specimen where all important features such as invasion depth, lymphovascular invasion, grade of differentiation and tumor budding can be assessed and thereby give the clinician solid information to base further treatment strategies on. However, ESD is considered technically challenging, with a long learning curve and it can be time-consuming. It has a higher risk of complications such as perforation and bleeding compared to EPMR [11]. To date, there is no clinical evidence concerning the need for hospitalization and when to begin food ingestion after a routine colorectal ESD. In Japan, with the largest experience of colorectal ESD, a routine ESD procedure is followed by nill per mouth on postoperative day one and a hospital stay of at least 2–4 days [12, 13]. Small European series also report inpatient care for two to three days with an initial fasting period after an ESD procedure [14].

This strategy could be questioned since new postoperative protocols for colorectal surgery stress the importance of early food intake and mobilization. In addition, unnecessary hospitalization leads to increased health care costs. Therefore, we investigated the feasibility of colorectal ESD as an outpatient procedure in a consecutive series of colorectal ESDs.

## Materials and methods

Based on the indications proposed by the Colorectal ESD Standardization Implementation Working Group and Colorectal ESD/EMR Guidelines established by the Japan Gastroenterological Endoscopy Society [15], all patients admitted to the Endoscopic Unit at Danderyds Hospital, Stockholm, Sweden with an early colorectal neoplasm larger than 20 mm, a local recurrence after earlier endoscopic treatments or where the position of the lesions or the endoscopic surface pattern did not make it suitable for EMR treatment, underwent colorectal ESD. Tumors showing endoscopic signs of deep submucosal invasion were referred for surgical resection.

Colonic cleansing was performed at home using 2000–3000 ml of polyethylene glycol (PEG) solution on the day before the treatment and 1000–2000 ml of PEG the day of the procedure. The patients came to the endoscopic unit fully prepped. No prophylactic antibiotics were administered. The ESD procedure was performed with a transparent cap (D201-11804,12704; 4 mm Olympus) fitted endoscope (GIF-H180 J, GIF-1TH190, PCF-190AI, Olympus, Tokyo Japan). Needle knives specially designed for ESD with minor modifications to the diathermy tip (Dual knife, KD-650Q, Olympus, Tokyo Japan) were used for the ESD procedure with a high-frequency generator (ERBE, Elektromed-VIO300D, Tubingen, Germany). When bleeding or vascular structures were encountered, hemostasis with a coagulation forceps (Coagrasper FD411-QR, Olympus, Tokyo Japan or SB junior, Sumitomo, Tokyo Japan) were used. Conscious sedation using midazolam and alfentanil was started with 2 and 0.5 mg doses intravenously, respectively and if required. Additional doses were administrated during the procedure on the endoscopists assessment. Hyoscine butyl bromide 10 mg was administered intravenously during the procedure if needed.

After the ESD procedure, patients were observed for 2–4 h and discharged if no significant symptoms such as increased abdominal pain or discomfort were seen. During this time-period, they were allowed to eat. Patients were admitted for in-hospital observation if their clinical status deteriorated after the procedure or if a procedural complication was anticipated.

Resected specimens were pinned with needles on a specimen plate, and the lesion and specimen size were measured. Subsequently, the specimens were immersed in 10% formalin and sectioned serially in 2 mm intervals for histological evaluation. Vienna classification was used to classify the colorectal specimens. En bloc resection was defined as resection in one piece of tissue for the whole lesion. Microscopically R0 resection was defined as no tumor cells in lateral or vertical resection margin whereas R1 resection was defined as lateral and/or vertical resection margin with tumor cells. Microscopically RX resection was used when the resection margin could not be fully assessed due to diathermy effects on the tissue.

### Complications and follow-up

Complications were defined as immediate or delayed. Perforations during the procedure were defined as complete, when serosa or intraperitoneal tissues was visualized, or partial, when fibers in the muscularis propria were incised, but no complete perforation occurred.

### Statistics and ethics

Data are presented as median (range), mean ± SD or true numbers as appropriate. Patient charts were assessed for any readmission within 30 days. The study protocol follows the Helsinki Declaration and was approved by the hospital institutional review board.

## Results

**Fig. 1:**
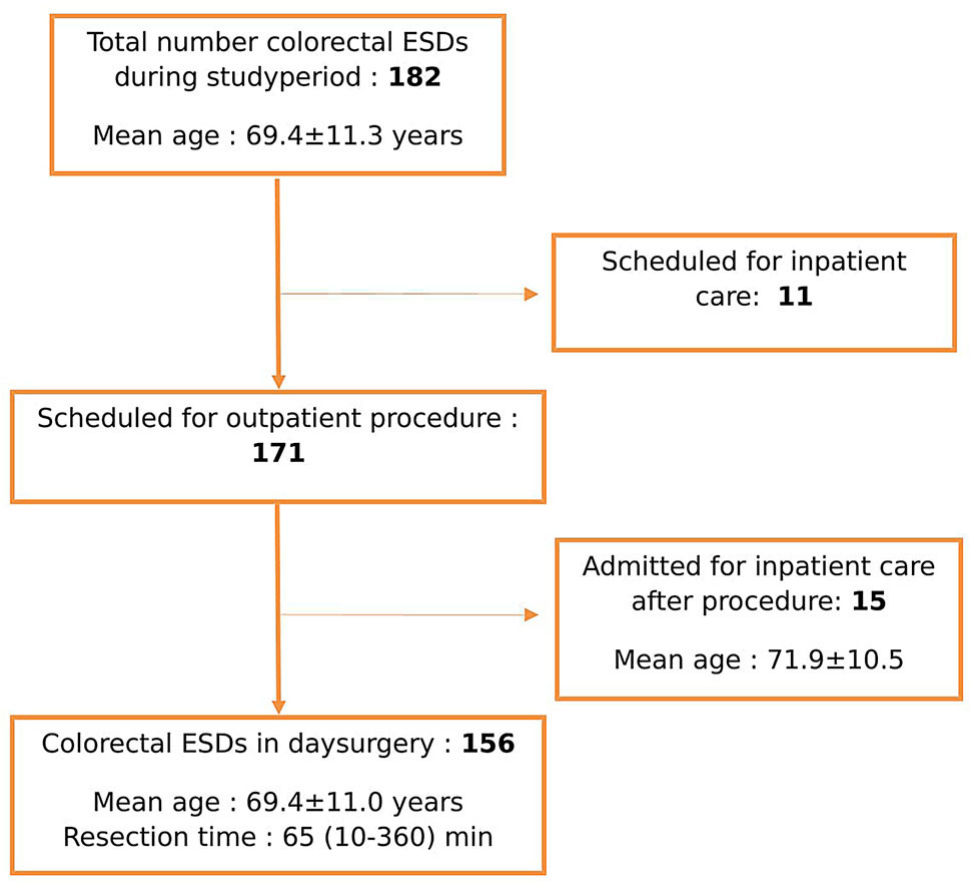
Consort flow diagram

During the study period, April 7, 2014 to December 31, 2015, a total of 182 colorectal ESDs were performed on 171 persons. Eleven patients were scheduled for an inpatient procedure. The reasons were living more than 100 km from hospital (3 patients), distal lesion requiring general anesthesia for adequate pain relief (3 patients) and age or co-morbidities that disabled patient to manage bowel preparation at home (5 patients). Patients on anti-platelet and/or anti-coagulant drugs were included in the study with a treatment criteria of PT-INR < 1.8. Anti-platelet drugs were discontinued 3–5 days before the procedure. Fifteen cases were initially planned as day surgery but were admitted to the hospital after the procedure at discretion of treating physician. Of these 15, one had an intraprocedural bleeding during rectal ESD that was not possible to manage endoscopically. He underwent emergent transanal endoscopic microsurgery (TEM) procedure. Postoperative course was uneventful. Eight had a transmural perforation of the bowel wall treated with clips and antibiotics. One of these eight patients required a laparoscopic wedge resection of the cecum postoperative day one after a cecal ESD procedure due to local peritonitis and free abdominal air. The postoperative course was uneventful. When comparing the 15 patients admitted to hospital after their colorectal ESD with the study cohort the former had a longer procedure time (median 170 (10–300) minutes), larger lesion size (median 45 (20–81) mm) and lesions were localized in cecum and ascending colon to a higher extent (73 vs 38%). Median length of stay was 1 [1–3] day. The remaining 156 cases out of a total of 182 were done in day surgery and these make up the study cohort (Fig. 1).

The sex distribution was 75 (48%) men and 81 (52%) women. Mean age was 69.4 ± 11.0 years. Distribution of the lesions is presented in Table 1. Endoscopic tumor characteristics are presented in Table 2. Of the flat lesions, a total of 83 were classified as lateral spreading tumor (LST) granular and 26 as LST non-granular. The most common indication for ESD in the cohort was a lesion size >20 mm (141 patients (90.3%)). Other indications were rectal carcinoid (one patient), lesions reaching the dental line (two patients), lesion extending into the ileocecal valve (one patient), LST non-granular lesions (three patients) and where position of the lesion made en bloc EMR hard to perform (eight patients).

**Table 1:** Location of included lesions

Location	Numbers	Percent (%)
Cekum	30	19.2
Ascending	29	18.6
Transverse incl flexures	24	15.4
Descending	3	1.9
Sigmoid	17	10.9
Rectum	52	33.3
Anal canal	1	0.6
	156	100

**Table 2:** Endoscopic characteristics of included lesions

Morphology (Paris classification)	Numbers	Percent (%)
0-Isp	10	6.4
0-Is	4	2.6
0-IIa	131	84.0
0-IIa + IIc	1	0.6
0-IIa + Is	9	5.8
Submucosal tumor	1	0.6
	156	100
NICE classification		
Type 1	32	20.5
Type 2	121	77.6
Type 3	0	0
Unclear	3	1.9
	156	100

Of the 156 cases, 152 (97.4%) were completed with ESD technique. Two (1.3%) cases were finished with snaring after mucosal incision and some submucosal dissection. One (0.7%) case ended as a piecemeal resection and one (0.7%) case was aborted due to difficulties establishing a submucosal plane. Median lesion size was 28 (10–120) mm and median resection time was 65 (10–360) min. Intraprocedural complications were seen in 14 (9.0%) cases. Three were transmural small perforations and 11 cases of injury to the muscular layer without transmural perforations. All these injuries were treated with endoscopic clips. There were no bleeding complications that could not be managed with endoscopic techniques. All patients had a clinical examination after 2–4 h and were discharged with no significant clinical findings. They were informed to contact the endoscopy unit or emergency department in case of abdominal pain, fever or rectal bleeding. All three patients with a transmural perforation were put on peroral antibiotics for 10 days. Another 3 patients with an incision in the muscular layer were put on peroral antibiotics at the endoscopists discretion.

The histological results of the lesions are presented in Table 3. Of the 7 people with adenocarcinoma, 3 had invasion <1000 micrometers into the submucosa and no lymphovascular invasion and the resections were considered curative. Three had subsequent surgical resection, one because of invasion >1000 micrometers and 2 with invasion <1000 micrometers but lymphovascular invasion. The remaining patient had a synchronous colon cancer with liver metastasis and was treated with palliative intent.

**Table 3:** Histopatholgy of included lesions

Histopathology	Numbers	Percent (%)
Tubular adenoma low & high grade dysplasia	48	30.8
Tubulovillous adenoma low & high grade dysplasia	63	40.4
Serrated adenoma	36	23.1
Neuroendocrine tumor	1	0.6
Superficial adenocarcinoma	7	4.5
Other	1	0.6
	156	100

R0 resection was seen in 129 (82.7%) of the cases. Rx was seen in 25 (16.0%). One case was done with a piecemeal resection, and one case was aborted during the procedure. Both these cases are assigned R1. Fifteen of the cases with Rx have had a repeat colonoscopy with no endoscopic sign of recurrence. Seven patients are scheduled for a colonoscopy within the coming 6 months. Three patients with Rx not be followed further, due to high age. The high rate of Rx with a normal follow-up endoscopy stresses the importance of having an adequate margin to make pathological assessment accurate.

All patients’ medical records were reviewed 30 days after the procedure. In the Stockholm area, some 80–90% of the family doctors and 6 out of 7 hospitals share the same computerized medical records and these records were reviewed to ensure that we did not exclude medical attention sought at another hospital/primary health care center. A total of 8 patients had been in contact with the Danderyd Hospital within the 30-days period, see Table 4. Three were admitted for observation, two with abdominal pain where a CT scan showed a local thickening of the bowel wall and small infiltration of extraluminal gas suggestive of microperforation or coagulation syndrome. They were treated conservatively with antibiotics. One patient required a blood transfusion. The other five patients were discharged from the emergency department after a normal clinical workup (blood tests and CT scan in case of abdominal pain). No patient required surgery related to the ESD-treatment during the follow-up. Of note is also that none of the patients who had a perforation or injury to the muscular layer during their ESD seen or readmitted to hospital during the first 30 days.

**Table 4:** Description of patients with postESD clinical event requiring medical attention

Patient	ESD location	Clinical event	POD#	In hospital care	Treatment
1	Cecum	Hematochezia + abdominal pain	1	Yes 4 days	Blood transfusion, antibiotics
2	Cecum	Abdominal pain	6	No	–
3	Ascending	Abdominal pain	1	Yes 3 days	Antibiotics
4	Ascending	Abdominal pain	3	No	–
5	Sigmoid	Abdominal pain	7	No	–
6	Rectum	Hematochezia	1	No	–
7	Rectum	Hematochezia	8	Yes 2 days	Observation
8	Rectum	Hematochezia	1	No	–

## Discussion

ESD is a technically challenging procedure with a long learning curve. There are currently no studies that have demonstrated that ESD can be performed as day surgery. Length of stay after various surgical procedures has become shorter during the past decades. In Sweden, 31% of the laparoscopic cholecystectomies and 78% of the inguinal hernia repairs were done as day surgery in 2014 [16]. The introduction of Enhanced Recovery After Surgery (ERAS) concept in colorectal surgery has shortened length of stay after colorectal resection stressing the importance of early oral food intake (on the same day of surgery) and early mobilization. The median length of stay after a colonic resection in a unit that adhere to the ERAS concept is now 2–3 days [17–19]. These data made us question the strategy to keep, often elderly, patients that undergo ESD fasting and in hospital for several days. In Japan, patients undergoing ESD are hospitalized for 5–6 days. In a Japanese study of 382 patients, the mean hospital stay after ESD was 5.3 days. With a clinical pathway focusing on early discharge including oral food intake on day 2 in uncomplicated cases, the mean hospital stay was decreased to 3.4 days [11]. Also, in a European setting, ESD patients are treated with an initial nill per mouth and hospitalization for 2–3 days [14]. In our study, we scheduled 171 out of 182 patients (94.0%) as day surgery, with a postESD observation for 2–4 h in the endoscopy unit. Of the 171 patients scheduled as day surgery cases 156 were done as day surgery cases (91.2%).

The two most common complications after ESD are bleeding and perforation, both can happen either acutely or delayed. In our series, we had a perforation rate of 1.9% (transmural injuries) which increased to 9.0% when injuries to the muscular layer were included. All injuries were treated endoscopically and none of the patients had any complications of the treatment. It is difficult to compare perforation rates between studies as different definitions of perforation are used (transmural injuries of injuries to the muscularis propria). In one of the largest ESD cohorts published, comprising 1111 colorectal ESD procedures, intraprocedural perforations were seen in 4.9% and delayed perforations in 0.4% [20]. Most intraprocedural perforations, however, are small and, if recognized, can be treated endoscopically with clips.

In our material 4 out of 156 cases developed a delayed bleed (2.6%). Intraprocedural bleeding problems were managed with available endoscopic hemostatic devices in all patients. One of 4 patients in our series with delayed bleed required blood transfusion. Delayed bleeding has been reported in 4.3% after colorectal ESD up to 7 days after the procedure [21].

Eight out of the 156 cases done as day surgery seeked medical attention within 30 days of the ESD procedure (5.1%). Five of these 8 patients were discharged on the same day after a medical examination including a combination of computed tomography, blood tests and clinical examination. Three patients (1.9%) needed inpatient care. Four of the 8 patients seeked medical attention postoperative day 1, and the other 4 seeked medical attention postoperative day 3–8. Of the 8 patients who sought medical attention within 30 days, 5 had abdominal pain which could possibly be a sign of an electrocoagulation syndrome. It is thought to be caused by an electrocoagulation injury to the bowel wall. This may lead to a transmural burn causing abdominal pain and fever within a few days after the injury. Electrocoagulation syndrome is thought to occur in about 1% of all EMR. A recent review states that Electrocoagulation syndrome is more common after ESD than EMR, occurring in up to 9% of colorectal ESD cases [22]. The electrocoagulation injury to the bowel wall has a benign course in most cases. All five cases in our series with delayed abdominal pain had a benign course.

The present study is a prospective ESD series from a single institution. Weaknesses of the study are that it is a single center study, patients included were more likely to have a lesion that was easy to treat with ESD and that patients where the procedure was technically challenging or time-consuming were more likely to be admitted to hospital and thus excluded from the cohort. However, even if including those patients, serious complications were rare with one patient requiring laparoscopic surgery for perforation and one patient requiring an emergency TEM for bleeding control. The other admitted patients were treated conservatively with antibiotics and were discharged the next day. Our data suggest that a large proportion of the patients having a colorectal ESD can safely be discharged the same day.

When defining an uncomplicated ESD several factors probably needs to be considered. Factors such as submucosal fibrosis, intraprocedural bleeding and access to the lesion might be of importance. All these factors could influence the risk of injury to the muscularis propria. Procedure time is a proxy variable that might be suitable. Median procedure time for day surgery cases in the present study was 65 min whereas median time for patients admitted to hospital after their procedure was 170 min. Further studies are needed in this field.

In the present study, we aimed to evaluate the efficacy and safety of colorectal ESD procedures in a day surgery setting in a tertiary referral center in Sweden. This is one of few European colorectal ESD series that include a large proportion of lesions in cecum and ascending colon (38%). We have demonstrated that it is feasible and safe to perform uncomplicated colorectal ESD in a day surgery setting, when patients receive verbal and written information about the postoperative predicted course. This information should also include information about symptoms of possible complications (bleeding, delayed perforation and electrocoagulation injury).

In this series, an experienced Japanese endoscopist (T.O) was full time backing up the all ESD procedure to maintain quality control and keep the complication rates to the standards of eastern countries.
